# Supplementary material for: The relationship between confidence and gaze-at-nothing oculomotor dynamics during decision-making
Source: PLoS One. 2025 Jul 7;20(7):e0310231. doi: 10.1371/journal.pone.0310231 (PMC12233307; doi:10.1371/journal.pone.0310231)
Supplement: S1 File — (PDF) [file pone.0310231.s001.pdf]

Supplementary Figure 1. A-B. For the reverse Look-At-Nothing (rLAN) experiment**.** Probability of selecting the Right image as a function of the ratio of time spent looking Right over looking Left images during the Stimulus-On (A) and during the Stimulus-Off (B) intervals of the rLAN experiment. Logistic regression fit for each individual participant and grand average (in black). **C-D**. Participants' sigmoid slope (β_1) parameter for each participant of the rLAN experiment, during the SOn (C) and SOff (D). Statistical significance was reported as (* P<0.05). **E.** Comparison of the distribution of the participants' sigmoid slope (β_1) and intercept (β_0) parameters between the Stimulus-On (SOn) and Stimulus-Off (SOff) for the sLAN experiment. **F-J.** Same as **A-E**, but for the sLAN experiment. **J.** Comparison of the distribution of the participants' sigmoid slope (β_1) and intercept (β_0) parameters between the Stimulus-On (SOn) and Stimulus-Off (SOff) for the sLAN experiment. **K.** Pearson correlation index between the reported confidence and the observation time, for each participant of the rLAN experiment. **L.** Same as K, but for the sLAN experiment.

Supplementary Figure 2. **A.** Group average β−Coefficients of a GLM regressing the observation time devoted to the selected image vs the indices of Confidence (C), Reward (R), Interest (I), Complexity (Cm), and interactions: RxI, RxCm, IxCm reported at the end of each trial, for the rLAN experiment. Group effects on Stimulus-Off are reported marginally for Confidence (C) (t-test; SOff-UD P=0.070), although the statistical relationship follows the same pattern of the LAN experiment, and for Reward (R), (t-test, SOn P=0.030). **B**. Correlation between observation time of the selected target until decision time and reported confidence. **C.** Same as A for the sLAN Experiment. No statistical significance is reported in this case.

Supplemental Figure 3. **A.** Distribution of #CoT and fCoT (right and left insets) for two typical participants and group average (left to right), during the rLAN Experiment, calculated during three intervals: Stimulus-On (SOn), Stimulus-Off Until Decision (SOff-UD), Stimulus-Off Post Decision (SOff-PD). **B.** Same as A, but for the sLAN Experiment.

Supplemental Figure 4. A. Mean and standard error of the Pearson correlation index between #CoT during SOn, SOff-UD and SOff-PD Intervals and confidence during the rLAN experiment. **B.** Same as A, but for the sLAN Experiment. **C.** Mean and standard error of the Pearson correlation index between fCoT during SOn, SOff-UD and SOff-PD Intervals and confidence during the rLAN experiment. **D.** Same as C, but for the sLAN experiment.

List of Contexts:

1. Which food brand would you rather be the commercial face of?
2. You are going to spend two days without...
3. You are going to travel twenty minutes by...
4. Which sense would you rather lose because of an accident?
5. Your roommate plays this instrument for three hours daily...
6. You are alone on an island and only have...
7. During a trip in the wild, would you rather urinate in?

8. You are stung by a non-dangerous…
9. Which animal would you be rather caged with?
10. You need to clean a big pile of dirt with only...
11. You drink a non-poisonous beverage made with...
12. As a quality control inspector, you are going to work for a company that produces.
13. As your lifelong project, you are writing a story about...
14. Which object would you rather get stolen?
15. You will never eat again…
16. You will have an ever lasting...
17. It is snowing, you wear only...
18. In an amusement park, you ride...
19. Tomorrow morning, you are going to be woken up by...
20. Where would you rather go on vacation with your family?
21. You have to clean weekly...
22. Which creature would you rather fight to save the world?
23. Which species would rather breed?
24. What would you rather take to a warzone?
25. Where would you rather stay for a week?
26. What scent would you rather prefer?'
27. What would you rather lose?
28. What's your favorite sacred animal?'
29. Which item would you rather get stolen while traveling abroad?'
30. Who would you rather talk to?
31. What would you rather have a nightmare with?
32. Which documentary would you rather watch?
33. Which obsession would you tolerate better for your partner?
34. Which company would you rather be CEO of?
35. What would you rather take when traveling 500 years into the future?
36. What would you rather take when traveling 2000 years into the past?
37. As a president, what would you promote?
38. What would you rather store in the case of famine?
39. Which inheritance would you like better?
40. Which winter sport would you rather try without a helmet?
41. What would you rather breakfast prior to running a marathon?
42. What would you take with you for a dangerous trip into the wild?
43. Where would you hide your favorite necklace?
44. Your favorite relative reincarnates as a...
45. Where would you prefer to spill coffee before an interview?
46. What's your favorite vehicle for sightseeing?
47. Where would you rather have lunch nearby?
48. Both animals have a terminal disease, who do you euthanize?
49. For the sake of our planet, what would you rather eliminate?
50. Where would you rather spend a night alone?
51. Where would you rather work to tour around the world?
52. As an engineer, what would you rather produce?
53. What would you rather have at home?

54. What would you rather eat to get you through your day?
55. Which free membership would you prefer?
56. What would you rather find in the forest?
57. What would you do if you get the list of correct answers before the exam?
58. You are locked in, what would you use to open the door?
59. Where would you rather date?
60. What would you rather have in a post-nuclear world?
61. Which injured animal would you stop the car to help at night?
62. Where would you prefer to discover a new life form?
63. What would you rather drink after brushing your teeth?
64. What would you rather eat during camping?
65. Who would you rather help?
66. What would you rather give to a poor person?
67. Which one would you save from extinction?
68. Which one would you rather have next to your house?
69. You will have an unlimited supply of
70. As a part of the Non-profit Fundraising Campaign, you organize
71. Which extreme sport would you rather try?
72. What would you rather wear during a long-distance cycling tour?
73. You bought a 100$ mystery box from eBay. What would you rather find in the box?
74. You are on campus, where would you rather study for your finals?
75. It is late at night, where would you rather be stuck?
76. What would you rather overexpress in your social media profile?
77. A close friend surprises you with a gift...
78. What would you rather find at the beach?
79. What would you rather have access to while having the flu?
80. As an entrepreneur, your successful business idea for a small town would be...

The list of image-stimuli presented along with each context is provided in the kaggle repository, indexed by the number of context plus one:

https://www.kaggle.com/datasets/e0e229739e3b27f4452ee20579596dac59a54d9f5af93db299c972e69fce9657.

Models' posterior parameters per participant:

	Model 1					Model 2				Model 3			
#S	α_0	α_1	α_2	BIC	F	α_0	α_1	BIC	F	α_0	α_1	BIC	F
1	0.3279	0.1714	0.1794	24.3892	-4.5284	0.3293	0.1984	14.8790	-2.9144	0.1754	0.6944	11.5314	-5.1313
2	0.2809	0.4823	0.1467	-11.7472	-23.0109	0.3287	0.1475	-13.6460	-24.2603	0.5304	0.5892	-11.8283	-22.6724
3	0.2117	0.1484	0.3759	15.5516	-3.0964	0.2094	0.4033	17.4229	-1.3569	0.1401	0.7366	15.4194	-2.3560
4	0.3310	0.6005	-0.0058	-5.3874	-17.9960	0.3515	0.0980	-10.9557	-21.8650	0.6109	0.4988	-5.3382	-17.6742
5	0.3401	0.1721	0.0944	6.8652	-8.7968	0.3416	0.1243	8.5461	-7.1845	0.1753	0.5633	6.3665	-8.7388
6	0.2243	0.1083	0.2315	14.9101	-3.5444	0.2304	0.2275	17.0501	-1.6527	0.1461	0.6334	14.0609	-3.2366

7	0.3662	-0.1176	0.0840	-4.0977	-16.4220	0.3697	-0.1010	-1.8523	-14.9931	-0.1643	0.5864	-5.5998	-17.1402
8	0.3192	0.2102	0.1588	-28.6128	-35.8794	0.3476	0.1298	-26.6690	-34.5543	0.2782	0.6345	-27.4321	-35.0446
9	-0.0122	-0.0426	0.7663	16.6352	-3.4796	-0.0188	0.7617	18.9960	-1.3488	-0.0408	0.7410	18.9752	0.0003
10	0.0860	0.3638	0.5681	28.3613	5.2311	0.0844	0.6321	18.9960	4.0590	0.3632	0.7080	30.5064	7.4397
11	-0.06125	0.0097	0.8598	14.3672	-4.7587	-0.0627	0.8608	25.2974	-2.7011	0.0217	0.7526	15.6213	-2.1782
12	0.3459	-0.1363	0.1833	10.6615	-6.4349	0.3442	0.1757	16.7777	-4.5965	-0.1308	0.7172	8.7124	-7.2243
13	0.4085	-0.0413	-0.4943	-17.5109	-26.7093	0.4061	-0.4924	12.4476	-25.3298	0.0512	0.2071	-18.1143	-26.9562
14	0.0064	0.1970	0.6528	29.1415	2.4498	0.0057	0.6724	-15.2132	3.7591	0.1808	0.6690	30.4747	7.3190
15	0.4839	0.1236	-0.1351	6.6356	-9.0029	0.4950	-0.1534	29.9161	-7.2678	0.2279	0.6411	0.9276	-12.5832
16	0.2179	0.0396	0.1874	1.3004	-12.4626	0.2207	0.1861	8.7779	-10.6170	0.0595	0.5218	2.8713	-11.0991
17	0.1240	0.2041	0.4444	16.5201	-3.1463	0.1147	0.4780	3.6425	-1.7116	0.1326	0.6774	14.9329	-2.6120
18	0.2933	0.2166	0.0796	1.7935	-12.2410	0.261	0.1502	3.4983	-10.8612	0.1400	0.5655	2.1452	-11.6174
19	1.1710	0.4499	-1.3643	-14.9585	-25.4761	1.2505	-1.4308	-14.2374	-25.1605	0.7802	0.4884	-28.4050	-35.7860
20	0.2577	0.3527	0.2188	2.4544	-12.0221	0.2788	0.2259	3.0061	-11.4451	0.3922	0.6102	2.4815	-11.4543
21	0.4733	0.1737	-0.1031	16.2347	-2.6447	0.4902	-0.1040	17.9109	-1.0401	0.2443	0.6533	7.3329	-7.9409
22	0.6640	-0.0346	-0.3058	-5.3937	-17.7254	0.6618	-0.3079	-3.0715	-15.9087	0.0513	0.6162	-12.6365	-22.9237

Suppl. Table 1. Posterior parameters per participant obtained for the LAN experiment

	Model 1					Model 2				Model 3			
#S	α_0	α_1	α_2	BIC	F	α_0	α_1	BIC	F	α_0	α_1	BIC	F
1	0.1990	-0.0920	0.4188	11.8387	-5.5943	0.1979	0.4133	14.1062	-3.8377	-0.0774	0.7410	11.0486	-5.1464
2	0.3870	0.2485	-0.1739	-20.4518	-29.3494	0.4296	-0.1950	-18.6892	-28.1261	0.4134	0.4583	-21.2024	-29.7432
3	0.4633	0.0895	0.0142	2.11258	-12.6071	0.4677	0.0147	4.2283	-10.5162	0.1140	0.7590	-2.3798	-15.4545
4	-0.0175	0.0489	0.7629	47.0098	14.4560	-0.0181	0.7666	49.4272	16.8542	0.0496	0.7333	49.5037	17.4805
5	0.07247	0.2356	0.8293	13.7082	-4.4031	0.0412	0.6341	16.1038	-2.2365	0.0235	0.6982	16.0606	-1.9925
6	0.0920	0.1279	0.4926	12.7596	-5.1704	0.0941	0.5010	14.6467	-3.2876	0.01307	0.6303	14.7086	-2.9600
7	0.0129	0.2041	0.5917	-2.2415	16.4892	-0.0049	0.6348	-0.8241	-15.0736	0.1956	0.6152	0.0805	-13.3970

Suppl. Table 2. Posterior parameters per participant obtained for the LAS experiment

	Model 1					Model 2				Model 3			
#S	α_0	α_1	α_2	BIC	F	α_0	α_1	BIC	F	α_0	α_1	BIC	F
1	0.2255	0.0902	0.4627	15.2859	-3.5006	0.2456	0.4351	16.9971	-1.2430	0.1055	0.7687	16.5046	-2.2601
2	-0.0747	-0.0336	0.6110	-32.2554	-40.3038	-0.0750	0.6123	-29.9950	-38.4423	-0.0389	0.4872	-30.5940	-37.8714
3	0.2805	-0.1940	-0.0097	-9.8026	-21.2016	0.2794	0.0028	-8.2630	-19.7352	-0.1924	0.4640	-8.9067	-20.3192
4	0.0813	-0.1746	0.5294	-3.3267	-12.9089	0.1187	0.4740	-1.9614	-14.7045	-0.1822	0.6541	-1.0283	-14.4688
5	0.1295	-0.2053	0.5394	1.5941	1.6282	0.0905	0.6363	2.9635	-11.5138	-0.1677	0.7217	3.4023	-11.0147

6	0.3298	-0.2793	0.2435	21.8534	-5.0610	0.3029	0.2853	22.4261	2.4961	-0.2455	0.6926	21.7019	1.9622
7	0.0617	0.2932	0.4413	12.1280	1.5823	0.3242	0.1630	10.9873	-5.5438	-0.5471	0.5446	13.5555	-3.5994
8	0.0.0860	0.3638	0.5681	22.8355	-14.4237	0.3284	0.1957	21.7435	1.5659	-0.4331	0.6279	22.2969	2.0780
9	-0.0625	0.0097	0.8598	1.4964	5.22311	0.0493	0.5174	2.8211	-13.3937	0.0681	0.6940	0.0993	-13.2426

Suppl. Table 3. Posterior parameters per participant obtained for the rLAN experiment

5

	Model 1					Model 2				Model 3			
#S	α_0	α_1	α_2	BIC	F	α_0	α_1	BIC	F	α_0	α_1	BIC	F
1	0.0200	0.2034	0.5545	25.2952	1.3910	0.0174	0.5715	27.2005	2.7779	0.1451	0.6171	26.6184	5.1369
2	0.0565	-0.2291	0.6636	36.6112	10.3215	0.0652	0.6238	38.6371	11.5615	-0.3165	0.8152	38.1727	13.0344
3	0.0602	0.0714	0.5167	26.9223	3.4642	0.0601	0.5189	29.3256	5.0695	0.0594	0.7426	24.9453	4..5934
4	0.1764	0.1986	0.0990	21.3098	-0.0083	0.1896	0.0872	22.4390	1.3786	0.2912	0.4490	16.2723	-1.8134
5	0.1700	-0.7037	0.2701	5.1489	-10.9431	0.1715	0.2473	6.7968	-9.7220	-0.2839	0.6604	-0.9707	- 13.8354
6	0.0516	-0.1676	0.4230	1.0420	-15.2009	0.0492	0.4192	2.5897	-13.5665	-0.1279	0.5595	-0.4367	- 13.8512
7	0.1473	0.4391	0.1981	0.0173	-14.6284	0.1463	0.2189	1.3199	-13.9873	0.3955	0.6159	-8.3773	- 19.0768
8	0.0019	0.0594	0.5542	27.0981	2.9304	0.0021	0.5553	29.5203	4.5042	0.0626	0.5612	29.5524	7.6632
9	0.188	-0.4196	0.6032	-3.5824	-18.9268	0.0202	0.5713	-2.6095	-18.3227	-0.5183	0.7494	-5.0503	- 16.5969
10	0.0504	0.2472	0.5386	20.9172	-0.8510	0.0492	0.5580	22.0064	0.3074	0.2142	0.6911	20.6292	1.3015
11	0.1054	0.2472	0.5386	0.6230	-14.1229	0.1128	0.2496	2.8918	-12.8622	-0.6689	0.8063	-0.7536	- 13.6142

Suppl. Table 4. Posterior parameters per participant obtained for the sLAN experiment

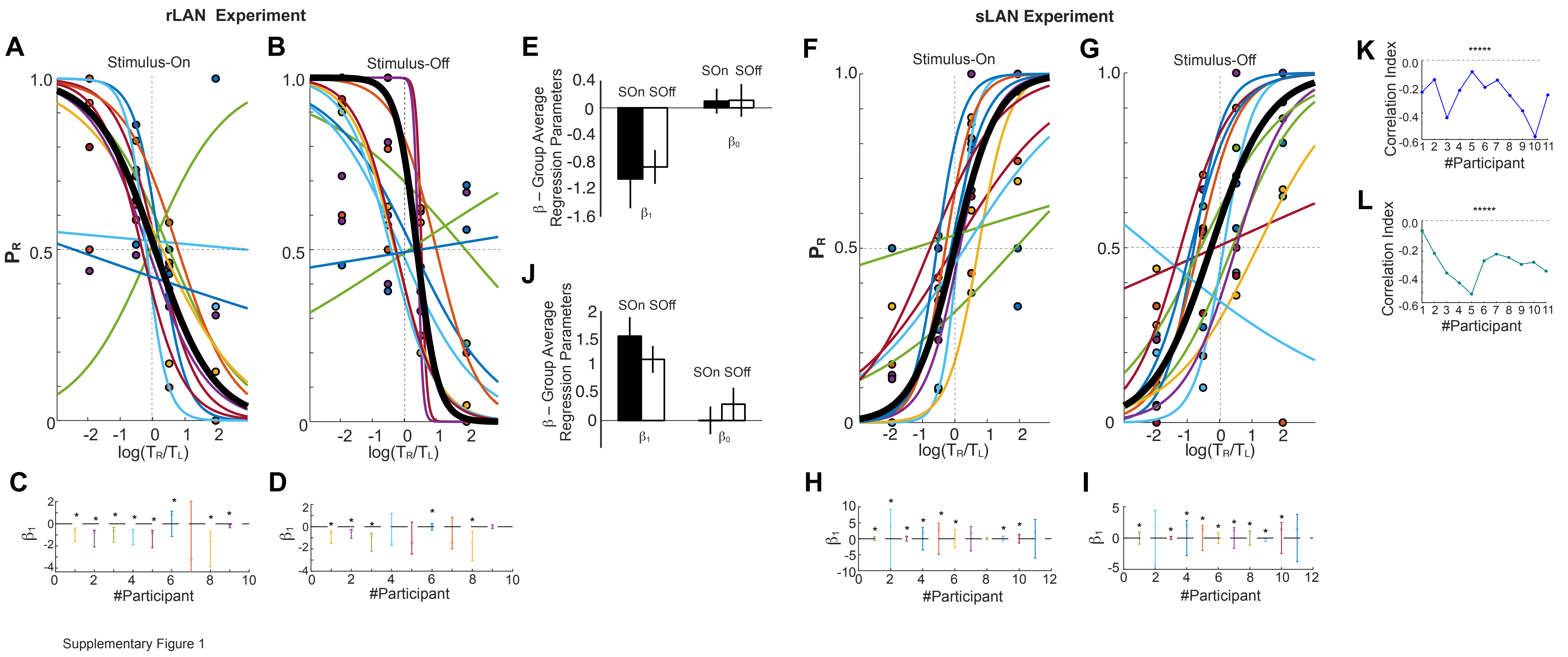
rLAN Experiment
sLAN Experiment
A
Stimulus-On
B
Stimulus-Off
E
β – Group Average Regression Parameters
SOn SOff
β₁
β₀
J
F
Stimulus-On
G
Stimulus-Off
K
Correlation Index
#Participant
L
P_R
log(TR/TL)
C
D
H
I
β₁
#Participant
Supplementary Figure 1

Suppl. Fig. 1

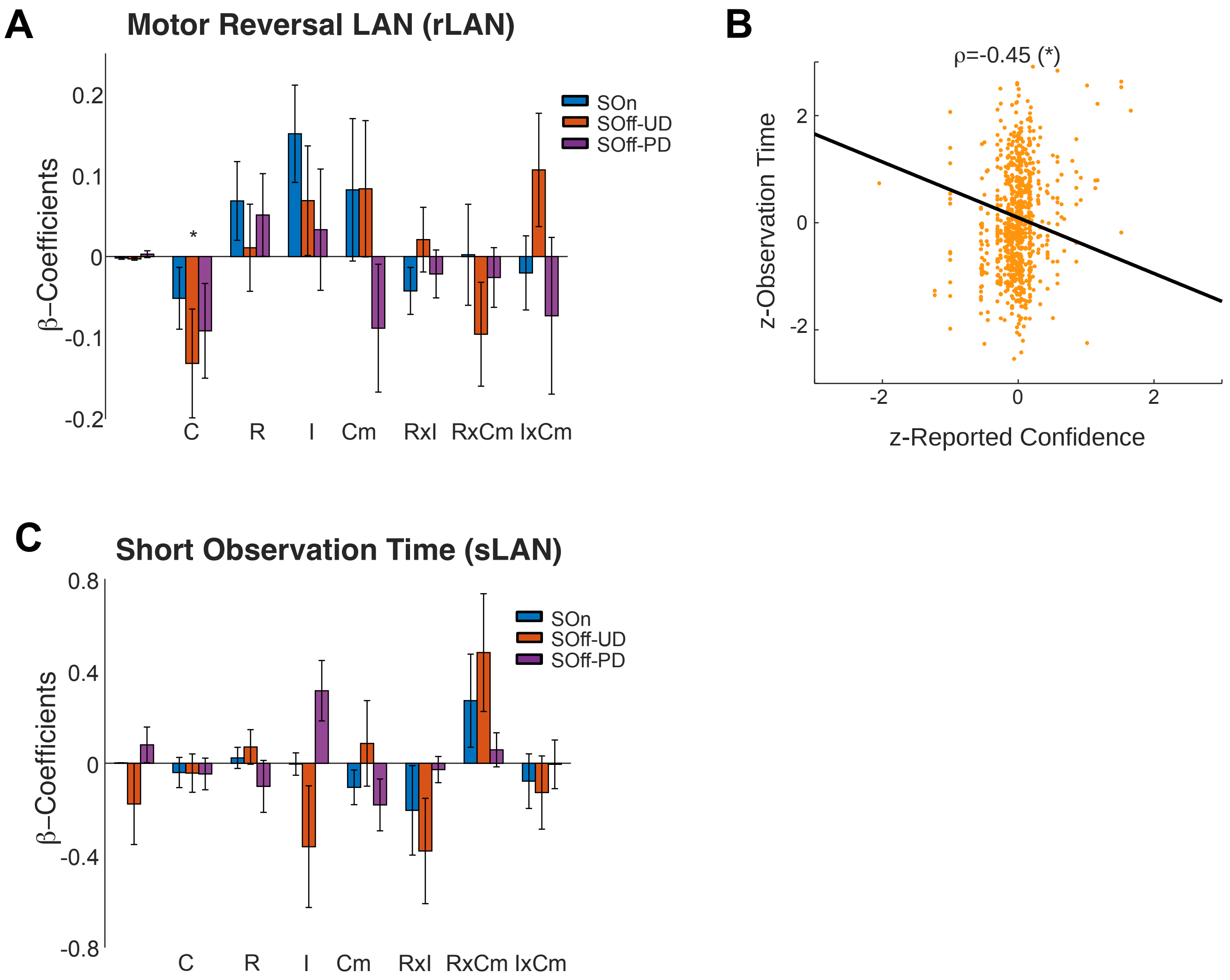

Supplementary Figure 2

Suppl. Fig. 2

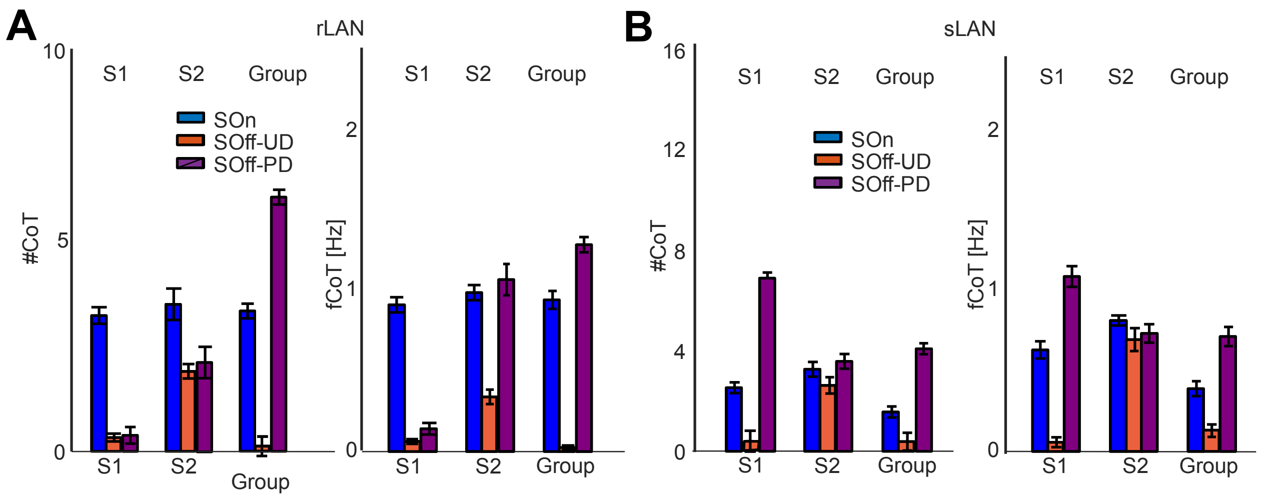
A
rLAN
10
5
0
#CoT
S1
S2
Group
SOn
SOff-UD
SOff-PD
2
1
0
fCoT [Hz]
B
sLAN
16
12
8
4
0
#CoT
SOn
SOff-UD
SOff-PD
2
1
0
fCoT [Hz]

Suppl. Fig. 3

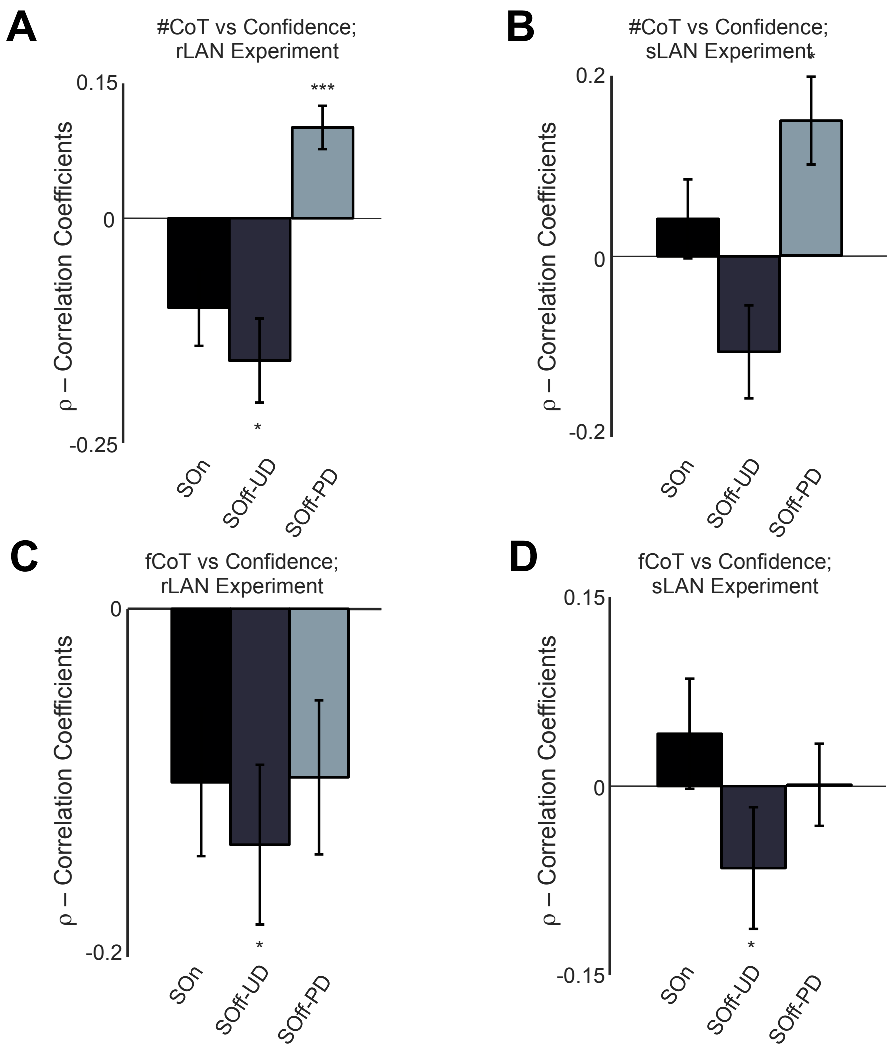

Supplementary Figure 4

Suppl. Fig. 4
